# Evaluation of Sustainability of a Prune Production Process from Farm to Fork Approach based on Thermodynamic Principles and Actual Operational Data

**DOI:** 10.1002/gch2.202100071

**Published:** 2021-10-15

**Authors:** Fatemeh Nadi, Arif Hepbasli

**Affiliations:** ^1^ Department of Agricultural Machinery Mechanics Azadshahr Branch Islamic Azad University Shahid Rajaee St. Azadshar 49617‐89985 Iran; ^2^ Department of Energy Systems Engineering Faculty of Engineering Yasar University Bornova Izmir 35100 Turkey

**Keywords:** conversion industries, exergy, food security, plum waste, prunes, renewable energy, sustainability

## Abstract

The main objective of the present study is to investigate energy consumption, exergy and greenhouse gas (GHG) emissions from prune production in both the garden and plant sectors. Both energy and exergy analysis methods are used while some sustainability indicators such as the cumulative degree of perfection (CDP) and the renewability indicator (RI) as well environmental aspects are considered. The analysis is based on the actual operational data. The high energy consumption, exergy and GHG emissions are related to the post‐harvest and the factory operations. Natural gas is determined to be the most effective input to energy consumption, exergy and GHG emissions in the whole process of producing prunes. Based on the sustainability indicators used, the agricultural operation of the plum production process is partially renewable while the factory operation of the prune production process is highly non‐renewable. In cases where the production process of prunes includes the use of renewable energy and plum waste, CDP increases from 0.32 to 2.88 and RI from ‐2.16 to 0.65. The use of renewable sources in producing one ton of prune annually reduces GHG emissions by 362.55 tons and energy consumption by 7.45 TJ worldwide. The use of plum waste would also produce 402.8 TJ of energy per year.

## Graphical abstract

The present study investigates prunes production, showing how the high energy consumption, exergy, and greenhouse gas emissions relate to the post‐harvest and the factory operations. Natural gas is the most impactful input in the whole process of producing prunes. The use of biogas instead of natural gas in prune production reduces energy consumption to 0.01 of custom consumption.

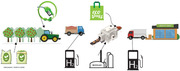

## 1 Introduction

Fruits have become the eighth produced commodity in the world, and Iran with producing 359 176 tons of plums per year is the fifth largest producer of plums in the world after China, Romania, Serbia, and Chile.^[^ ^1^ ^]^ In recent years, plums have been recognized as a health‐promoting food. Also, prune (dried plum) is known for its laxative effect. Studies attribute the laxative effect of prune to the presence of phenolic and sorbitol and its high fiber content. Drying of plums has been done for thousands of years near the Caspian Sea, the same place where European plums originated. Prunes have spread throughout Europe with the migration of different cultures and civilizations.^[^ ^2^ ^]^

Agriculture and the food industry are among the largest consumers of energy in the world. Identifying the share in energy consumption at each stage may help presented appropriate energy consumption strategies and appropriate decisions to reduce energy consumption.^[^ ^3^ ^]^ Performing energy input–output analysis of a crop production will not consider the quality of inputs and energy conversion as an essential key element.^[^ ^4^ ^]^ For overcoming this issue, one can use exergy analysis, which is a concept applying the first and second laws of thermodynamics, as a particularly useful tool in addressing the impact of energy use on the environment. This is a suitable technique for more efficient use of energy resources because it can identify situations, waste types, and actual amounts of exergy losses.^[^ ^5^ ^]^ With the CEXC approach, farmers and conversion industries can improve production efficiency. The cumulative degree of perfection (CDP) and renewability indicator (RI) for each stage of the production process can be calculated through the CEXC method and the overall productivity ultimately can be improved.

As far as some studies done in the area are concerned, Karakaya and Özilgen^[^ ^6^ ^]^ surveyed the process of making tomato paste and estimated the share of the agricultural sector in the required energy at 19.58%, with the share of this sector in the greenhouse gas (GHG) emission at 48.8%. Sogut et al.^[^ ^7^ ^]^ considered the high temperature difference between steam and tomato paste as the cause of exergy losses in the tomato paste factory and proposed an automatic control system to reduce the pressure drop due to steam accumulation in the tank before entering the evaporation unit. According to Özilgen and Sorguven,^[^ ^8^ ^]^ in the production of sunflower, olive, and soybean oil, agriculture had the largest share of energy consumption, exergy, and GHG emissions from the whole process. Bozoglan and Hepbasli^[^ ^9^ ^]^ examined the olive oil refining factory and found that the maximum exergy was related to the boiler, distillation system, and steam generator, pH control system and reverse osmosis to prevent corrosion of boilers while utilization of more efficient pumps was proposed to increase the factory exergy efficiency. In a malt beverage factory, exergy analysis showed that the pasteurization and packaging unit was responsible for 50% of the system inefficiency. The integration of packaging and pasteurization units was proposed to improve the energy efficiency of the system.^[^ ^10^ ^]^ In the exergy analysis, CDP of production of strawberry flavored yogurt from farm and livestock operations to production and packaging operations was estimated to be 0.037. By replacing hydraulic sources to generate electricity, the CDP value increased to 4.6.^[^ ^11^ ^]^ Pelvan and Özilgen^[^ ^12^ ^]^ investigated the production process of instant, black, and ice tea and concluded that the tea production process was highly nonrenewable and even with the production of activated carbon and hydrogen from instant tea waste, it was still nonrenewable.

Drying, cleaning, and packaging units had the largest share in energy, exergy, and GHG emissions throughout the black tea production process.

Food loss or food waste significantly reflects the inefficient use of valuable resources of food production, which reduces natural resources and environmental pollution, as well as undermining food security. The economic impact of food waste is also significant.^[^ ^13^ ^]^ Food security is one of the important solutions to improve living standards. But it is not possible without access to renewable energy. Increasing attention is being paid to the installation and use of renewable energy sources in the agricultural sector in several countries with the aim of reducing global greenhouse gases and food security.^[^ ^14^ ^]^ Based on the literature review done, the process of producing dried fruits, considering food security, and the sustainable development factor, has not been addressed so far to the best authors’ knowledge. This research attempts to perform a thermodynamic analysis during the production process of the prune, using the approach from the farm to the fork to identify inefficient energy stages of processes and point out the required new technology for improving them.

In this regard, this study aims at contributing to the existing literature as follows: i) the energy resources usage was evaluated by the CExC approach, ii) renewable values (CO_2_ emission, CDP, and RI) of various processes were determined as production sustainability indicators based on CExC, and iii) based on the CEXC approach, the effects of using renewable energy and plum waste on the improvement of CDP and RI factors as sustainability indicators were investigated.

## 2 Experimental Section

**Figure 1:**
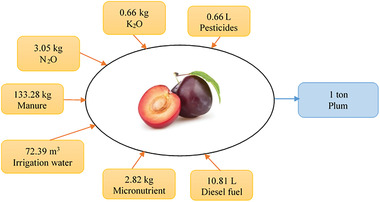
Inputs used to produce 1 ton of fresh plums.

In this study, the production process of prune was investigated based on the first and second laws of thermodynamic. Thermodynamic analysis was performed according to the inputs used to produce 1 ton of fresh plums in the garden and 1 ton of prune slices in the factory. According to **Figure** **1**, the inputs used to produce 1 ton of fresh plums are about 152.83 L of diesel fuel, 9.26 L of pesticide, 43.14 kg of nitrogen, 9.37 kg of phosphorus, 39.92 kg of micronutrients, and 1883.45 kg of manure, and 1023 m^3^ of water with a yield of 14.13 tons of plum per hectare, as extracted from the work of Shaghozayi and Nadi.^[^ ^15^ ^]^

**Table** **1** shows the inputs used to produce prune slices in the factory. The production process of prune slices from fresh plums includes washing, pitting, slicing, drying, and packaging.

**Table 1:** Inputs used in drying manufacture to produce prunes

Operations	Inputs	Value
Agriculture	Fresh plum	1000 kg
Washing	Electricity	3 kWh
	Water	2 m^3^
Pitting	Electricity	5 kWh
Slicing	Electricity	4.5 kWh
Drying	Natural gas	50 m^3^
	Electricity	7.5 kWh
Packaging	Electricity	0.8 kWh
	Bag	0.78 kg
Transportation	Petrol	20 l
	Outputs	
	Prunes	112 kg
	Plum pits	26.5 kg

## 3 Theoretical and Mathematical Formulations for Exergy Analysis

Exergy can help identify and explain the benefits of sustainable energy and technologies, meaning that there is a link between exergy and sustainability.^[^ ^16^ ^]^ Sustainability has also been combined with exergy to design environmentally friendly energy conversion systems.^[^ ^17^ ^]^ Energy analysis according to the first law of thermodynamics is a traditional method for estimating different energy conversion processes. The first law of thermodynamics is only about energy conversion and does not give information about component or system inefficiencies in a detailed manner. However, the second law of thermodynamics is related to the quantity and quality of energy.^[^ ^18^, ^19^, ^20^ ^]^ In the second law of thermodynamics, the quality of energy is determined by exergy.^[^ ^19^, ^21^ ^]^ Exergy analysis leads to a greater and deeper understanding of the process, as well as new ideas for improving the system.^[^ ^5^ ^]^ The second law presents insight into irreversibility, measures energy losses, and suggests the necessary measures to minimize it. Simply put, one can describe exergy as the ability to produce useful work depending on the state of the environment. Mass, energy, and exergy balance equations to produce 1 ton of prune slices can be written as follows^[^ ^18^, ^19^, ^21^ ^]^

Mass balance
(1) $$\begin{array}{c} \sum m_{\text{in}}=\sum m_{\text{out}} \end{array}$$
Energy balance
(2) $$\begin{array}{c} \sum {\left(mh\right)}_{\text{in}}-\sum {\left(mh\right)}_{\text{out}}=\sum Q_{k}-W \end{array}$$
Exergy balance

Exergy of material is the maximum work that can be extracted from a material if it achieves thermally, mechanically, and chemically an equilibrium with its surroundings through reversible processes. Exergy balance may be written as follows

(3) $$\begin{array}{c} \sum {\left(mb\right)}_{\text{in}}-\sum {\left(mb\right)}_{\text{out}}+\sum Q_{k}\left(1-\frac{T_{0}}{T_{k}}\right)-W=X_{\text{loss}} \end{array}$$

where *m* is the mass, *Q* is the heat, *T* is the temperature, *h* is the specific enthalpy, *W* is the work, the subscript *k* indicates the heat sources and *b* is available exergy flow of the product, which can be calculated from the following equation^[^ ^20^ ^]^

(4) $$\begin{array}{c} b=b^{\text{ch}}+b^{\text{th}} \end{array}$$

where *b* ^ch^ and *b* ^th^ are the chemical and physical exergies of the products, respectively. The amount of available flow (*b*) for the plum and prune is described in Table A.3

(5) $$\begin{array}{c} b=-T_{0}S=R_{u}T_{0}\underset{i}{\sum }y_{i}\ln(y_{i}) \end{array}$$

where *T*_0_ is the ambient temperature, *s* is the specific entropy, *R*_u_ is the universal gas constant, and *y_i_* is the molar fraction.

Cumulative degree of perfection (CDP) is defined as a ratio between the chemical exergy of a product and the total exergy of all raw materials and fuels used during production as follows^[^ ^20^ ^]^

(6) $$\begin{array}{c} \text{CDP}=\frac{{\left(mb\right)}_{\text{product}}}{\sum {\left(m\text{CExC}\right)}_{\text{raw}\;\text{materials}}+\sum {\left(m\text{CExC}\right)}_{\text{fuels}}} \end{array}$$

Berthiaume and Bouchard (1999) defined Szargut's exergetic approach by developing the concept of the cumulative exergy consumption (CE*_x_* C), net exergy consumption (CNE*_x_*), and reconstruction work (*W*_r_). In this regard, net exergy cumulative consumption (CNE*_x_*) was defined as the difference between CE*_x_* C and chemical exergy content of the final product (*X*_p_), as given below

(7) $$\begin{array}{c} {\text{CNE}}_{x}={\text{CE}}_{x}C-X_{p} \end{array}$$

The following relation was also proposed for the energy utilization of nonrenewable sources^[^ ^22^ ^]^

(8) $$\begin{array}{c} W_{r}≅{\text{CNE}}_{xp}+{\text{CNE}}_{xwaste} \end{array}$$

where CNE*_x_*_p_ is the consumption of nonrenewable energy sources and CNE*_x_*_waste_ is the waste treatment process.

For being an energy source renewable, the useful work obtained by that source must be larger than the reconstruction work. In this study, prune slices were considered as the final product and the renewability indicator was adapted based on the following equation^[^ ^22^ ^]^

(9) $$\begin{array}{c} \text{RI}=\frac{\left(X_{p}-W_{r}\right)}{X_{p}} \end{array}$$

where *X*_p_ is the useful work derived from the product. If the maximum working potential of the product is extracted through reversible processes, *X*_p_ is equal to the exergy of the product.

RI has four modes:

(RI = 1): Completely renewable process.
(0 < RI < 1): The process is somewhat renewable.
(RI = 0): Production and reconstruction work is equal.
(RI < 0): Nonrenewable process.

## 4 Results and Discussion

Food production is a process that consumes high amount of exergy while emitting the most CO_2_. Exergy analysis offers a realistic view of the energy loss to the environment and irreversibility in the process. Cumulative energy consumption (CEnC) and cumulative exergy consumption (CExC) are specific thermodynamic indicators associated with food production. In this research, the energy consumption and exergy values along with CO_2_ emissions from prune slices production were investigated to manage and save energy. To assess the process sustainability in addition to GHG emissions, CDP and RI were calculated, and according to the obtained results, a strategy for reducing energy consumption and exergy and hence increasing sustainability was investigated.

In this context, the results were comprehensively discussed below in terms of cumulative energy consumption, cumulative exergy consumption, GHG emissions, assessing the sustainability of the process, utilizing renewable resources as a new strategy, and scenario of using plum waste as biomass.

### 4.1 Cumulative Energy Consumption

**Figure 2:**
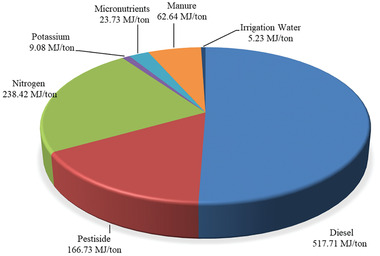
Energy of inputs used to produce 1 ton of plums.

**Figure** **2** shows energy values of inputs to produce 1 ton of fresh plums. The CEnC value for production of 1 ton of plums was obtained 1.02 GJ. Based on some studies, to produce 1 ton of horticultural products such as sweet cherries, sour cherries, and apples, consumption values were 6.90, 7.24,^[^ ^23^ ^]^ and 2.03–2.36 GJ,^[^ ^24^ ^]^ respectively. Comparison of the results shows that energy consumption for plum production is less than that for sweet cherry, sour cherry, and apple production.

Diesel fuel was energy‐consuming input in the production of plums. The share of diesel input in total cumulative energy consumption was 50.58%. Because ≈77% of diesel fuel is used in the production of plums for irrigation,^[^ ^15^ ^]^ the effect of the irrigation system on the CEnC is very important. Taki and Yildizhan^[^ ^25^ ^]^ obtained similar results for greenhouse cucumber cultivation. They also reported that for the irrigation system, diesel engines used a lot of energy to extract water from deep wells. Considering that more than half of the energy consumption for plum production is related to diesel, reducing diesel consumption will specifically reduce energy consumption. After diesel fuel, nitrogen fertilizer (23.29%) has the greatest impact on CEnC. Yildizhan and Taki^[^ ^26^ ^]^ also mentioned that fertilizer and diesel were energy‐consuming inputs to tomato production.

The calculations were done by considering that approximately every 8929 kg of fresh plums yields 1000 kg of prunes. **Table** **2** illustrates that 42.20 GJ of energy is consumed to produce each ton of prune slices, of which 9.14 GJ are related to agriculture and horticulture while the remainder relates to factory and transportation operations. In other words, the shares of energy consumption in horticultural and factory operations are 21.66% and 78.34%, respectively. Among the inputs consumed during the production process of prune slices, natural gas with 53.36% has the highest share in the energy consumption. The results of Pelvan and Özilgen^[^ ^12^ ^]^ also indicated that in the preparation of black tea and instant tea, the highest energy consumption was due to drying process. For olive, sunflower, and soybean oil industries, the share of energy consumption of agricultural operations is more than that of the oil processing and production operations. Energy consumption values for processing olive, soybean, and sunflower oil were 1.07, 1.18, and 1.64 GJ t^–1^, respectively.^[^ ^8^ ^]^ Based on a study done by Karakaya and Özilgen,^[^ ^6^ ^]^ in the tomato paste industry the share of energy consumption in the agricultural sector of tomatoes was low while the energy required to produce 1 ton of tomatoes was 4.75 GJ. Baking energy consumption of bread in different countries was estimated to be 1.41–46.87 GJ t^–1^ of bread depending on the type and amount of ingredients used in bread and the energy source of the bread baking oven.^[^ ^27^ ^]^ In the wheat bread industry, the share of wheat agriculture for bread production was less than the share of processing operations (which included flour preparation, dough preparation, bread baking, and packaging).^[^ ^28^ ^]^ Comparison of the results of this study with other studies shows that among different conversion industries, drying operations are more energy‐consuming compared to other food processing units.

**Table 2:** CEnC values for the prune production processes

Operations	Inputs	CEnC [MJ t^–1^]	Share of each operation [%]
Agriculture	Plum	9139.23	21.66
Washing	Electricity	321.43	0.97
	Water	89.29	
Pitting	Electricity	535.71	1.278
Slicing	Electricity	482.14	1.14
Drying	Natural gas	22098.21	54.27
	Electricity	803.71	
Packaging	Electricity	85.71	1.10
	Bag	378.00	
Transportation	Petrol	8267.86	19.59

### 4.2 Cumulative Exergy Consumption

Performance analysis of processes using the second law of thermodynamics provides a logical approach to evaluation. Exergy analysis is a powerful tool in designing, optimizing, and evaluating the performance of energy systems to determine energy quality, compare different energy sources, and achieve maximum system performance. Because it allows all losses due to irreversibility to be quantified and examine the possible alternative ways of doing work that ultimately leads to minimal energy consumption.

**Figure 3:**
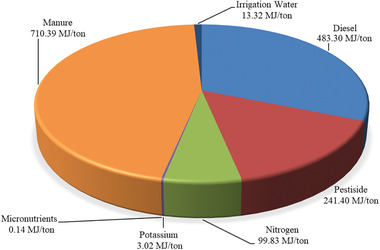
CExC for production of 1 ton of fresh plum.

Reducing the values for CExC can save natural resources for the next generation, as well as control GHG emissions and improve human quality of life. The CExC value for production of 1 ton of plum was estimated to be 1.55 GJ (**Figure** **3**). Since there are very few studies on the exergy of horticultural products in the available sources to the best of authors’ knowledge, the exergy of plum production was compared with other products.

The total CExC values for open field and greenhouse strawberry production were estimated to be 8.74 and 14.18 GJ t^–1^, respectively. The increase in CExC in greenhouse cultivation compared to open field cultivation was due to high electricity consumption in greenhouses.^[^ ^29^ ^]^

The total CExC value for 1 ton of irrigated and rainfed wheat production were 7.70 and 3.45 GJ, respectively. The reason for the high CExC for 1 ton of irrigated wheat from rainfed wheat production was the use of electricity as the main source to pump water from deep wells in the irrigated wheat production system.^[^ ^30^ ^]^ CExC was estimated at 9.40 GJ t^–1^ for apple,^[^ ^31^ ^]^ 4.65 GJ t^–1^ for greenhouse cucumber,^[^ ^25^ ^]^ 7.34 GJ t^–1^ for sunflower, 6.09 GJ t^–1^ for soybean, and 7.90 GJ t^–1^ for olive production.^[^ ^8^ ^]^ Comparison of the results shows that the CExC value of fresh plum production is less than other agricultural products.

In the production of fresh plums, the highest exergy consumption was related to animal manure (710.39 MJ) followed by diesel (483.30 MJ) and pesticide (241.40 MJ). **Table** **3** lists CExC values for the prunes production processes. Exergy required to produce 1 ton of prune slices from farm to fork approach was 51.00 GJ, of which 13.85 GJ t^–1^ of prune slices were used in horticultural operations, with the remainder in the factory. The share of horticultural operations in the total CExC was 27.16% and the remainder belonged to the factory operations. The largest share of exergy consumption in the production of prunes was related to the drying process (46.67%). Among the consumed inputs, natural gas with 46.56% of the total exergy was the most effective exergy input.

**Table 3:** CExC values of the prunes production processes

Operations	Inputs	CExC [MJ t^–1^]	Share of each operation [%]
Agriculture	Plum	13852.40	27.16
Washing	Electricity	5178.57	10.17
	Water	8.61	
Pitting	Electricity	23.92	0.05
Slicing	Electricity	19.37	0.04
Drying	Natural gas	23750.00	46.67
	Electricity	53.81	
Packaging	Electricity	0.61	1.07
	Bag	546.00	
Transportation	Petrol	7571.43	14.84

Based on the results reported by Pelvan and Özilgen,^[^ ^12^ ^]^ for the making of black tea and instant tea, the drying operation had the largest share of exergy consumption. CExC values in GJ ton^−1^‐product from farm to fork for olive oil production was 43.05, with 17.64 for sunflower oil, 45.26 for soybean oil,^[^ ^8^ ^]^ 16.6 for wheat bread, 14.7 for rye bread, 25.2 for hamburger bun,^[^ ^28^ ^]^ 43.53 for black tea, 3.99 for instant tea, 4.46 for ice tea,^[^ ^12^ ^]^ 74.65 for yogurt, and 16.75 for strawberry jam.^[^ ^11^ ^]^ Comparison of the results indicates that the making prune slices consumes more exergy than the process of making oil, tea, strawberry jam, and bread.

A comparison between the results of Figures 2 and 3 demonstrates that the values for CEnC and CExC are different. Because in exergy analysis, various energy sources with different quality can be analyzed. Considering that exergy is defined as a method for evaluating the quality of various resources,^[^ ^32^ ^]^ CEnC and CExC values can be different for each energy source.

### 4.3 GHG Emissions

**Figure 4:**
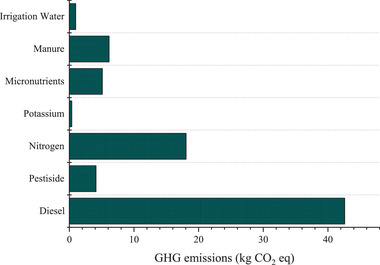
GHG emissions of inputs used to produce 1 ton of plums.

An environmental parameter, cumulative specificity (CCO_2_E), is parallelly used with other thermodynamic parameters to determine the GHG emissions during production. According to **Figure** **4**, 77.43 kg of CO_2_ was emitted from producing 1 ton of fresh plums in the garden. The majority of GHG emissions in the production of fresh plums were related to diesel, nitrogen, manure, and micronutrient fertilizers, respectively. The portion of other inputs was less than 5% in GHG emissions. Due to high fertilizer consumption, in addition to increasing the price of crops, groundwater pollution, and the toxicity of agricultural products cause air pollution, so it is crucial to control it.

The results of other researches showed that agricultural operation emits 164.9, 317.4, 756.8, 0.175, 0.3, 0.2, 0.51, 0.24, 0.025, 137.56 kg of CO_2_ into air to produce 1 ton of olives, sunflower, soybean,^[^ ^8^ ^]^ cucumber,^[^ ^25^ ^]^ wheat, rye,^[^ ^28^ ^]^ greenhouse strawberry, strawberry in the field open,^[^ ^29^ ^]^ tomatoes,^[^ ^26^ ^]^ and apple,^[^ ^31^ ^]^ respectively. It can be concluded that the production of oilseeds and tree fruits has the highest GHG emission value while GHG emissions to produce 1 ton of other agricultural products are estimated at less than 1 kg.

**Table** **4** shows that the production of 1 ton of prune slices emits ≈2072.17 kg CO_2_, of which 691.39 kg is related to horticultural operations and the remaining 1380.78 kg of CO_2_ emitted relates to factory operations. This means that plant with 66.63% has the largest share in GHG emissions. Drying unit with 37.74% of the total amount of CO_2_ emitted has the largest share in CCO_2_E. Natural gas input accounts for 37.64% of CCO_2_E, so modifying the drying process or using a renewable source instead of natural gas will significantly help reduce the CO_2_ emissions caused by the production of prune slices.

**Table 4:** CCO_2_E values for the prunes production processes

Operations	Inputs	CCO_2_E [kg CO_2_ eq]	Share of each operation [%]
Agriculture	Plum	691.39	33.37
Washing	Electricity	0.88	8.49
	Water	175	
Pitting	Electricity	1.47	0.07
Slicing	Electricity	1.33	0.06
Drying	Natural gas	779.91	37.74
	Electricity	2.21	
Packaging	Electricity	0.24	0.62
	Bag	12.60	
Transportation	Petrol	407.14	19.65

According to the farm‐to‐fork approach, through the production of 1 ton of strawberry jam, yogurt, black tea, instant tea, ice tea, wheat bread, rye bread, hamburger bun, olive oil, sunflower oil, soybean oil, the values of kg of CO_2_ emitted are 1005,^[^ ^11^ ^]^ 1917,^[^ ^11^ ^]^ 1733, 140, 267,^[^ ^12^ ^]^ 0.9, 0.07, and 1.1,^[^ ^28^ ^]^ 323, 492, and 0.860,^[^ ^8^ ^]^ respectively. The results show that the drying industry emits more CO_2_ than other agrifood industries.

### 4.4 Assessing the Sustainability of the Process

Exergy efficiency for producing crop was evaluated using CDP and IR. CDP is related to processes of nonrenewable energy sources and is defined as a ratio between output exergy and total input exergy.^[^ ^12^ ^]^ The amount of CDP in the production process is dependent on the chemical structure of the product and the cumulative exergy consumption. According to Equation (6), if the chemical exergy of the product or the availability of a product is large and the cumulative exergy consumption of the product is small, it can increase the CDP value of the product manufacturing process.

With a high value of CDP, the exergy losses of the production process are low and the renewability index of the production process is high, meaning that the product manufacturing process is more environmentally friendly.

CDP values to produce 1 ton of fresh plum and prune slices were determined by considering the values of inputs and exergy of plum and prune as an output factor. Based on the results, the CDP value for plum production is 1.71, while that for the whole making prune is obtained 0.32. The low amount of CDP in the making of prunes indicates the need to improve production techniques.

Other studies indicated that the CDP values for cucumber production were 0.23,^[^ ^25^ ^]^ 0.29 for field strawberries, 0.18 for greenhouse strawberries,^[^ ^29^ ^]^ 1.62 for tomatoes,^[^ ^26^ ^]^ 2.9 for irrigated wheat, 6.48 for dry wheat,^[^ ^30^ ^]^ 0.425 for black tea, 0.013 for instant tea,^[^ ^12^ ^]^ while wheat and rye cultivation in Turkey were estimated to be 3.73 and 4.96, and for wheat and rye agriculture in Germany were 11.26 and 10.46,^[^ ^28^ ^]^ respectively.

In the conversion industries, for making soybean oil, olive oil, and sunflower oil, they were 0.92, 0.98, and 2.36, respectively,^[^ ^8^ ^]^ and 0.036 for flavored yogurt^[^ ^11^ ^]^ were reported. Comparison of the results of the present study and other studies shows that the conversion industry sector significantly reduces CDP compared to the agricultural sector with the same product. The amount of CDP should also be reduced to less than 1, except for making sunflower oil. Because the amount of CDP is related to the chemical composition of the product, especially the amount of fat, and because the amount of fat in oilseeds is higher than other products, the CDP of oil products is higher than that of other processed products even after processing.

The RI was studied for explaining the share of renewable energy consumption in the plum production process. Few studies have used this index to analyze energy and exergy. The results showed that the RI for the production process of fresh plums is 0.42 and for prune slices is −2.16. RI for cucumber production process was estimated to be 3.32,^[^ ^25^ ^]^ for irrigated wheat was 0.65, for dryland wheat was 0.84,^[^ ^30^ ^]^ and for tomato was estimated to be 0.38.^[^ ^26^ ^]^

In the conversion industry, the RI for bread was −0.55,^[^ ^27^ ^]^ for black tea was −1.35, for instant tea was −31.30, and for ice tea was −610668,^[^ ^12^ ^]^ so it can be concluded that the process of producing fresh produce is somewhat renewable. While the processed product manufacturing process is strictly nonrenewable.

Because RI is essential for environmental protection as well as sustainable development and food security, in food production, in addition to the management of the agricultural sector, the management of food factories needs special attention. Most of the food is processed and highly nonrenewable. The use of new and sustainable technologies as well as moving toward new energies can reduce the use of fossil fuels and make the renewable indicator positive.

### 4.5 Utilizing Renewable Resources as a New Strategy

In many countries, the choice of right energy sources is debated. Many believe that in the long run there is a need for renewable energy sources and less polluting fuels. The development and use of sustainable resources, i.e., resources that meet current needs without compromising future needs, is becoming increasingly socially acceptable. Therefore, renewable energy sources are becoming an important alternative energy source in the agrifood sector. The use of renewable energy sources leads to increased sustainability, food security, production of environmentally friendly products, and reduction of dependence on fossil fuels being discharged. The more countries prioritize the use of renewable resources, the lower the cost of these resources. Therefore, such a transfer does not create an economic burden. In this section, the role of nonrenewable energy sources in the agricultural sector as a tool to increase sustainable food security is explained and technologies, policies, and opportunities in the use of renewable energy in the agricultural sector are reviewed and analyzed. Therefore, two scenarios were considered.

In the first scenario, it is assumed that electricity is supplied by wind energy, machines use liquid hydrogen (LH_2_), biogas replaces natural gas, and farmers use microbial fertilizers instead of chemical fertilizers, and then exergy analysis is performed again.

Wind‐electricity requires fewer fossil energy (0.0404 MJ) and produces less GHG (4.13 g CO_2_eq) per kWh of electricity generated. Therefore, the use of wind‐electricity is an important option against decarbonization.^[^ ^33^ ^]^ According to Ertürk and Özilgen,^[^ ^34^ ^]^ biogas can also be obtained from a wide range of wastes, including manure, slaughtered chicken feathers. The sustainability of the use of biogas in a process depends greatly on how these processes relate. Börjesson and Berglund^[^ ^35^ ^]^ estimated the amount of GHG emissions (21 g CO_2_eq) and the cumulative energy required (0.4 MJ) to produce each MJ of biogas. According to the results by Özilgen,^[^ ^36^ ^]^ energy and exergy and hence the amount of GHG emissions produced by microbial fertilizers be very negligible.

The energy required to produce LH_2_ fuel is 60 MJ kg^–1^ while the LH_2_ energy content is three times that of gasoline, and for each liter of gasoline, 0.34 L of LH_2_ is required. The LH_2_ exergy is 138 596 MJ kg^–1^.^[^ ^37^ ^]^ Considering that the LH_2_ density is 67.8 kg m^–3^, so the energy and exergy were obtained 1.38 and 3.24 MJ  L^–1^, respectively. Wind‐electricity exergy of 2.29 MJ kWh_el_^−1^ was estimated,^[^ ^38^ ^]^ and biogas exergy of 0.0345 MJ kg^–1^ biogas^[^ ^39^ ^]^ was estimated.

**Figure 5:**
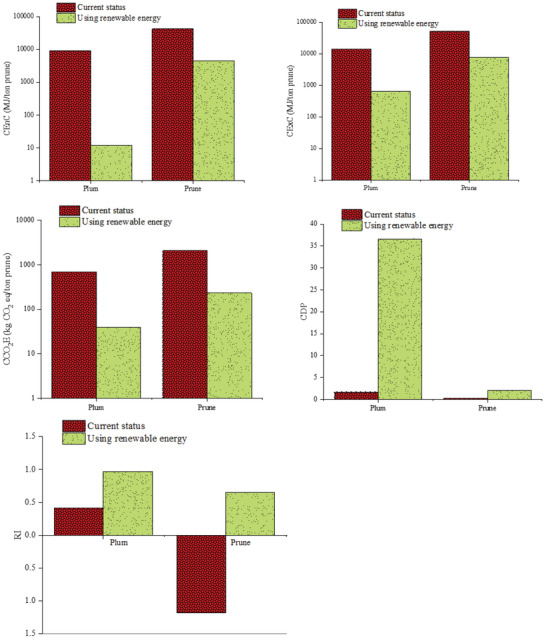
Effect of using renewable energy on the sustainability index of the production system of fresh plums and prunes.

**Figure** **5** shows the effect of the new scenario on the cumulative consumption of exergy and sustainability indicators, i.e., GHG emissions as well as CDP and RI in the process of producing plums and prunes. For the new case, CExC decreased for plum and prune production to 72.44 MJ t^–1^ plum and 7.80 GJ ton^−1^ prune, respectively. This result can confirm that the use of renewable resources can reduce the total exergy losses in the production of products and create a more sustainable system. The CDP value of plums increased from 1.71 to 36.62 and for prunes from 0.32 to 2.07.

Increasing the value of CDP compared to the earlier conditions means that in the new strategy, the input efficiency has increased. In the new case, the RI values for plum and prune production were 0.97 and 0.52, respectively, indicating that the new status is more renewable than before. These values represent a significant improvement in the production of environmentally friendly products.

As seen in Figure 5, the impact of using renewable resources on agricultural and horticultural operations is greater than factory operations. The reason for this is water consumption in the washing unit. As a result, it is suggested that the manufacturers of fruit and vegetable washing machines seek design methods for reducing water consumption in the washing unit.

Every year, a lot of time and money is spent in laboratories researching different dryers to reduce energy consumption. However, the results of this study showed that the use of biogas instead of natural gas while increasing the sustainability of the production process of dried products reduces energy consumption and the amount of GHG emissions to 0.01 of custom consumption.

### 4.6 Scenario of using Plum Waste as Biomass

**Figure 6:**
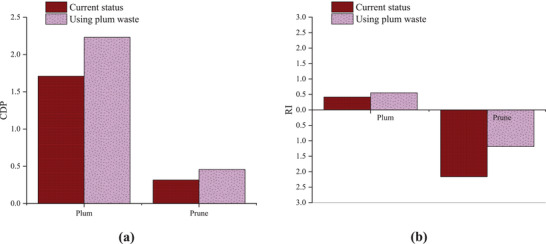
Effect of using plum waste on the sustainability index (CDP and RI) values of the production system of plums and prunes.

Discovering new and economically renewable sources is expanding. One of these renewable sources is agricultural residues. Agricultural residues, such as straw, fruit seeds, and molasses, fruit shells, nut shells stalks’ green leaves, are potential sources of renewable energy. Effective disposal of waste is an important part of environmental protection and its conversion into biomass is the most significant means of reducing the volume of waste compared to other methods of disposal. According to 2019–2020 statistics yearbook, 197 207 tons of prunes were produced annually in the world.^[^ ^32^ ^]^ The energy potential of plum pits is 15.2 MJ kg^–1^,^[^ ^31^ ^]^ each 8.929 kg of fresh plums gives 1 kg of dried plums, and so 834.1 TJ of energy can be produced annually from plum pits in the world. Bilgen^[^ ^40^ ^]^ estimated the plum pit exergy as 22.57 MJ kg^–1^ as biomass. Therefore, the use of plum residue for biomass production increases CDP from 1.71 to 2.23 for plum production and from 0.32 to 0.46 for prune production and RI from 0.0.42 to 0.55 for plum production and from −2.16 to −1.18 for prune production (**Figure** **6**a).

It is also possible to run both scenarios, namely, the replacement of fuels and chemical fertilizers and the production of biomass from the plum residues. If both scenarios are implemented together, energy consumption in the garden will increase the CDP from 1.71 to 47.48 and in the factory, it will increase the CDP from 0.32 to 2.88. RI also increases from 0.42 to 4.06 in the garden and from −2.16 to 0.65 in the factory (Figure 6b).

It can be concluded that the CExC approach is a useful tool for assessing the energy‐exergy flow of the agrifood industry and can help farmers and managers choose the best management method to optimize and enhance productivity. The results of this section showed that the use of renewable resources in agricultural activities, and especially in the processing plant would reduce exergy loss in production processes and save energy. The new hypothesis makes a significant contribution to sustainable food production methods and food security. Increasing investment in renewable energy resources can reduce the consumption of fossil fuels, environmental pollution, and ultimately improve human health.

## 5 Conclusion

Reducing dependence on fossil fuels and hence reducing CO_2_ emissions are among the goals of sustainable development policies and food security in the world. In this study, the process of making prune slices was evaluated using thermodynamic concept to determine management and to save energy. Through the CExC approach, the renewable status of the food making chain was assessed. Also, some sustainable production methods for production process of prunes were investigated. Main concluding remarks may be listed as follows:

1) Natural gas, nitrogen fertilizer, and diesel were the most effective inputs to energy consumption, exergy, and GHG emissions of prunes, indicating that this system is highly dependent on fossil resources.
2) The CDP value for prunes was estimated to be 0.32 and indicated a need to improve production techniques.
3) The RI values were obtained as 0.42 and −2.16 for plum and prune production systems, respectively. This factor showed that the production system of plums was partially renewable while prunes were completely nonrenewable.
4) Assuming replacement of microbial fertilizer with chemical fertilizer, LH_2_ with fossil fuel, and electricity supply by wind as a renewable energy source, the energy required to produce prune reduced from 42.20 to 4.42 GJ ton^−1^, with a decrease in the amount of GHG emissions from 2072.17 to 233.7 kg CO_2_eq t^–1^ prune_._ The CDP also increased from 0.32 to 2.07 and the RI from −2.16 to 0.52.
5) The first scenario reduced annually 362.55 tons of CO_2_ and saved 7.45 TJ of energy per ton prune. In addition, the use of plum pits as biomass produced 843.1 TJ of energy per year.
6) In case of using plum waste, the amount of CDP would increase from 0.32 to 0.46 and RI from −2.16 to −1.18.
7) Using both scenarios, the CDP value increased from 0.32 to 2.88 and the RI from −2.16 to 0.65.
8) Every year, a lot of time and money is spent in laboratories researching different dryers to reduce energy consumption. But the results of this study showed that the use of biogas instead of natural gas reduces energy consumption and the amount of GHG emissions to 0.01 custom consumption.

Performing energy‐based exergetic, exergoeconomic, and exergoenviromental analyses and assessments is recommended for future studies.

## Appendix

The chemical compositions of fresh plum and prunes are listed in **Tables** **A.1** and **A.2**, respectively. Calculation of plum availability flow of plum and prunes was performed according to Özilgen and Sorgüven.^[^ ^41^ ^]^ The availability flows of fresh plum and prune were obtained to be 3.39 and 16.13 MJ kg^–1^, respectively.

**Table A.1:** Chemical composition of plum^[^ ^42^ ^]^ and chemical exergy of component^[^ ^26^ ^]^

Chemical composition		Specific CExC	y_i_RT_0_ln(*y_i_*)
Carbohydrate	7.52%	25.95	−0.0005
Protein	1.30%	25.35	−0.0001
Fat	0.90%	37.17	−0.0001
water	86.88%	0.043	−0.0003
Fiber	1.50%	0	
Ash	1.90%	0	−0.0002

**Table A.2:** Chemical composition of prune^[^ ^43^ ^]^ and chemical exergy of component^[^ ^26^ ^]^

Chemical composition		Specific CExC	*y_i_RT*_0_ln(*y_i_*)
Carbohydrate	58.5%	25.95	−0.0008
Protein	2.8%	25.35	−0.0002
Fat	0.7%	37.17	−0.0001
water	25.0%	0.043	−0.0008
Fiber	8.1%	0	
Ash	5.0%	0	−0.0004

**Table A.3:** CEnC, CExC, and CCO_2_E coefficients of the inputs used to produce dried plums

Type of input	CEnC equivalent	CExC equivalent	CCO_2_E equivalent
Diesel	47.87 MJ L^–1[^ ^44^ ^]^	53.2 MJ kg^–1[^ ^20^ ^]^	0.0823 kg MJ^–1[^ ^45^ ^]^
Electricity	12 MJ kWh^–1[^ ^44^ ^]^	4.17 MJ MJ^–1[^ ^20^ ^]^	0.308 kg MJ^–1[^ ^46^ ^]^
Natural Gas	49.5 MJ m^–3[^ ^44^ ^]^	48.7 MJ kg^–1[^ ^20^ ^]^	0.853 kg MJ^–1[^ ^46^ ^]^
Gasoline	46.3 MJ L^–1[^ ^44^ ^]^	42.4 MJ L^–1[^ ^47^ ^]^	2.28 kg kg^–1[^ ^48^ ^]^
Irrigation water	1.02 MJ m^–3[^ ^49^ ^]^	2.6 MJ L^–1[^ ^20^ ^]^	0.192 kg m^–3[^ ^46^ ^]^
Piped water	0.005 MJ L^–1[^ ^50^ ^]^	0.29 MJ L^–1[^ ^51^ ^]^	0.0098 kg L^–1[^ ^51^ ^]^
Nitrogen fertilizer	78.1 MJ kg^–1[^ ^52^ ^]^	32.7 MJ kg^–1[^ ^20^ ^]^	5.917 kg kg^–1[^ ^46^ ^]^
Potassium	13.7 MJ kg^–1[^ ^53^ ^]^	4.56 MJ kg^–1[^ ^54^ ^]^	0.579 kg kg^–1[^ ^46^ ^]^
Micro	8.4 MJ kg^–1[^ ^55^ ^]^	0.05 MJ kg^–1[^ ^56^ ^]^	1.8083 kg kg^–1[^ ^57^ ^]^
Manure	0.47 MJ kg^–1[^ ^58^ ^]^	5.33 MJ kg^–1[^ ^41^ ^]^	0.0462 kg kg^–1[^ ^41^ ^]^
Pesticides	254.45 MJ L^–1[^ ^55^ ^]^	368.4 MJ kg^–1[^ ^51^ ^]^	6.3 kg kg^–1[^ ^51^ ^]^
Paper	10 MJ kg^–1[^ ^51^ ^]^	59.9 MJ kg^–1[^ ^20^ ^]^	1.88 kg kg^–1[^ ^51^ ^]^
Nylon	54 MJ kg^–1[^ ^51^ ^]^	78 MJ kg^–1[^ ^59^ ^]^	1.8 kg kg^–1[^ ^6^ ^]^

The CEnC, CExC, and CCO_2_E coefficients of the inputs used to produce prunes, which have been extracted from various sources, are given in Appendix A.3.

## Conflict of Interest

The authors declare no conflict of interest.

## Author Contributions

F.N.: Conceptualization, Methodology, Software, Formal analysis, Investigation, Resources, Writing‐ Original draft preparation, Investigation, A.H.: Reviewing and Editing.

## Data Availability

Research data are not shared.
